# Rapid identification of tsunamigenic earthquakes using GNSS ionospheric sounding

**DOI:** 10.1038/s41598-020-68097-w

**Published:** 2020-07-06

**Authors:** Fabio Manta, Giovanni Occhipinti, Lujia Feng, Emma M. Hill

**Affiliations:** 1 Earth Observatory of Singapore, Nanyang Technological University, Singapore, Singapore; 2 Asian School of the Environment, Nanyang Technological University, Singapore, Singapore; 3 Institut Universitaire de France, Paris, France; 4 Present Address: Université de Paris, Institut de Physique du Globe de Paris, CNRS, 75005 Paris, France

**Keywords:** Natural hazards, Seismology, Tectonics

## Abstract

The largest tsunamis are generated by seafloor uplift resulting from rupture of offshore subduction-zone megathrusts. The rupture of the shallowest part of a megathrust often produces unexpected outsize tsunami relative to their seismic magnitude. These are so called ‘tsunami earthquakes’, which are difficult to identify rapidly using the current tsunami warning systems, even though, they produce some of the deadliest tsunami. We here introduce a new method to evaluate the tsunami risk by measuring ionospheric total electron content (TEC). We examine two *M*_*w*_ 7.8 earthquakes (one is a tsunami earthquake and the other is not) generated in 2010 by the Sunda megathrust, offshore Sumatra, to demonstrate for the first time that observations of ionospheric sounding from Global Navigation Satellite System (GNSS) can be used to evaluate the tsunamigenic potential of earthquakes as early as 8 min after the mainshock.

## Introduction

‘Tsunami earthquakes’, as originally defined by Kanamori^1^, are events generating tsunami with larger amplitude than expected from their seismic magnitude. Most tsunami earthquakes are generated by high levels of slip on the shallow megathrust, which results in large seafloor uplifts and hence very dangerous tsunami. The shallow location of the slip—close to the subduction trench—means that the ruptures generating tsunami earthquakes are at significant distance from land-based monitoring networks, limiting our ability to quickly and accurately assess their magnitude and source parameters. Conventional approaches using various seismological methods^2–4^ or rapid inversion of GNSS (Global Navigation Satellite System) estimates of ground motion^5^ regularly encounter difficulties in accurately estimating the uplift of the seafloor and consequently fail in predicting the tsunamigenic nature of tsunami earthquakes. These events are generated in the relatively soft accretionary wedge and are characterized by slow rupture velocities, and therefore lower levels of seismic energy radiation^6,7^; they are thus only weakly felt by nearby populations, prohibiting natural warnings.

**Figure 1:**
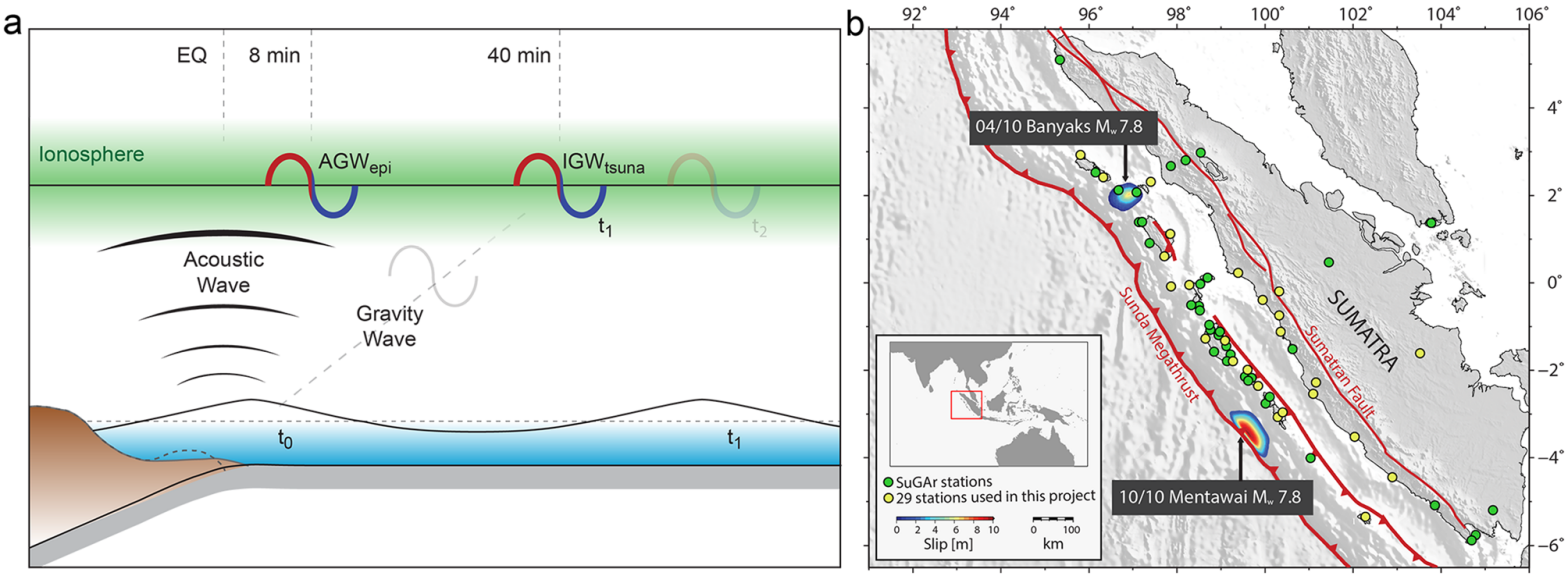
(**a**) Schematic of the two ionospheric signals associated with a tsunamigenic earthquake. At the time of the earthquake (t_0_), the sudden rupture of the fault causes an uplift of the sea floor and subsequently triggers a tsunami. Acoustic Gravity Waves (AGW_epi_) associated with the uplift propagate through the atmosphere and reach the ionosphere ~ 8 min after the rupture. The tsunami is an oceanic internal gravity wave; consequently, by a linear coupling, it produces an atmospheric Internal Gravity Wave (IGW_tsuna_). The IGW_tsuna_ reaches the ionosphere at time t_1_, ~ 40 min after the tsunami is initiated. (**b**) Main geological features of the Sumatran subduction zone. The contoured areas represent estimated coseismic rupture patches for the 6 April 2010 *M*_*w*_ 7.8 Banyaks earthquake in the north^19^, and the 25 October 2010 *M*_*w*_ 7.8 Mentawai earthquake in the south^20^. The yellow dots represent the SuGAr stations existing and available at the time of the events, while the green dots represent the current total extension of the SuGAr network.

In addition to seismic waves propagating within the solid Earth, tsunamigenic earthquakes can induce waves in the atmosphere, including the ionosphere. Firstly, the uplift of the seafloor at the source generates an acoustic-gravity pulse (AGW_epi_) that propagates through the atmosphere above the epicentral area (Fig. 1). This perturbation travels at the speed of high-atmospheric acoustic waves (~ 1,000 m/s) and reaches the ionosphere approximately 8 min after the event (Fig. 1)^8^. Due to the short response time, this ionospheric perturbation can provide critical information for rapidly assessing the tsunami potential. Secondly, if an ocean tsunami is generated, another perturbation later reaches the ionosphere. A tsunami is essentially an oceanic gravity wave; consequently, by linear dynamic coupling, it produces an atmospheric internal gravity wave (IGW_tsuna_), which reaches the ionosphere approximately 40 min after the event (Fig. 1)^9^. During the upward propagation, the generated AGW_epi_ and IGW_tsuna_ are both strongly amplified by the double effects of the exponential decrease of the atmospheric density ρ with altitude and the conservation of the kinetic energy ρv^2^, where v is the local speed oscillation induced by wave crossing. At ~ 300 km altitude in the ionosphere, the AGW_epi_ and IGW_tsuna_ are amplified enough to induce strong perturbations in the plasma density. These perturbations can be observed by ionospheric monitoring techniques, such as measurements of Total Electron Content (TEC) by GNSS (see^10^ for a review) or airglow cameras^11,12^. The TEC represents the number of electrons integrated along the ray-path between the GPS satellite and the station (see “Material and methods" for more details about TEC calculation). The signature of tsunamigenic earthquakes in the GNSS-derived TEC is consequently detectable in both the near-field by observing the AGW_epi_ directly related to the uplift of the seafloor^13,14^ and the far-field by observing the IGW_tsuna_ related to tsunami waves^9,11,15–17^.

**Figure 2:**
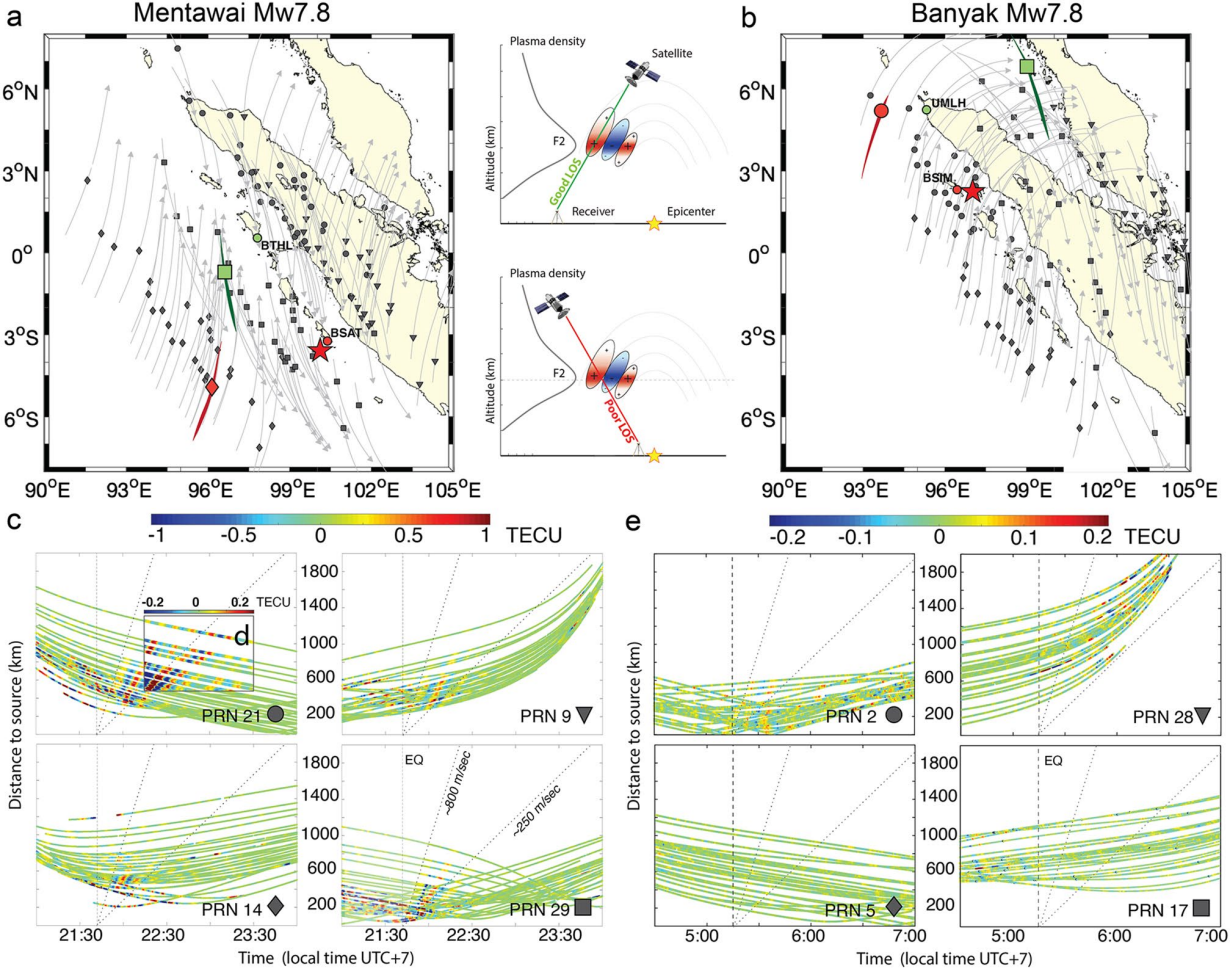
(**a**) Map of the distribution of ionospheric piercing points (IPPs) for the pairs of 29 GNSS stations and the four closest satellites at the time of the Mentawai earthquake (local time: 21:42:23 UTC + 7) (**b**) Map of the distribution of ionospheric piercing points (IPPs) for the pairs of 29 GNSS stations and the four closest satellites at the time of the Banyaks earthquake (local time: 5:15:02 UTC + 7). The IPPs are represented by four different symbols corresponding to different satellites; the symbols are located at positions along the trace of the IPPs corresponding to the event time. Note the bold traces highlighting the trajectory of the satellite-receivers pairs discussed in Fig. 3. The red stars represent the epicenter of the earthquakes. The cartoon of “Good LOS” (green) and “Poor LOS” (red) configurations is shown between **a** and **b**. (**c**) Hodochrones of the TEC perturbation observed by GPS satellites PRN 21, 9, 14, and 29. (**d**) Inset with color-scale 5 times smaller to highlight the gravity waves related to the IGW_tsuna_ recorded by PRN 21. (**e**) Hodochrones of the TEC perturbation observed by GPS satellites PRN 2, 28, 5, and 17. Color-scale in (**e**) is 5 time smaller than (**c**). Vertical dashed lines represent the time of the event. The gray dashed lines show the speed of the AGWepi (800 m/s) and IGWtsuna (250 m/s).

Based on the relation between the AGW_epi,_ which reaches the ionosphere approximately 8 min after an event, and the uplift of the seafloor, we investigate the potential of using GNSS observations of ionospheric TEC to discriminate tsunami earthquakes. We propose a technique to validate the risk estimated by conventional seismological methods, based on quantification of the intensity of the AGW_epi_, to discriminate the tsunamigenic nature of an earthquake. As a case study, we examine two *M*_*w*_ 7.8 megathrust earthquakes that occurred in 2010 along the Sumatran subduction zone, which were both recorded by the Sumatran GPS Array (SuGAr) (Fig. 2). Although both events had the same magnitude, and were issued similar tsunami early warnings (later canceled), the 6 April 2010 Banyaks earthquake^18,19^, did not generate a significant tsunami and caused only minor earthquake-related damage to local infrastructure. On the contrary, the 25 October 2010 Mentawai event produced a large tsunami—recorded within 10 min of the earthquake by the Mentawai GPS buoy—which claimed more than 400 lives from communities along the coastlines of the Pagai Islands^7,20,21^ (see “Material and methods" for a comprehensive description of the two events and the corresponding responses of tsunami warning systems).

Our analysis of the ionospheric response to these two events provides a unique natural experiment to test if the ionosphere is sensitive to the rupture properties of earthquakes and tsunami genesis, and whether GNSS-TEC observations are capable of identifying the events that have a high level of tsunami potential.

At the time of the Mentawai earthquake, 29 stations of the SuGAr network were in operation (Figs. 1b and 2a), while 25 stations were operating at the time of the Banyaks earthquake (Figs. 1b and 2b). In both cases, more than eight GPS satellites were visible in the sky, and we selected the four that better detected the events with comparable observation geometries. Cahyadi and Heki^22^ observed that changes of the penetration angles of the line-of-sight (LOS) with respect to the AGW_epi_ wave front propagating in the ionosphere can influence the amplitude of the detected signals. The authors referred to “good LOS” as the most favorable scenario for which the ionospheric piercing point (IPP) is located between the epicenter and the GNSS station (Fig. 2), in this configuration the LOS crosses mainly the positive part of the wave front returning a strong TEC signal. On the contrary they referred to “poor LOS” as the scenario for which the GNSS station is located between the epicenter and the IPP. With this second geometric configuration the LOS simultaneously penetrates the positive and negative parts of the anomaly, partially cancelling each other and consequently returning a weak signature in the GNSS-derived TEC. Moreover, as a consequence of the integrated nature of TEC along the station-satellite LOS, satellites at low elevation angles have a better detection capability^8^ (see Supplemental Figs. 1 and 2).

**Figure 3:**
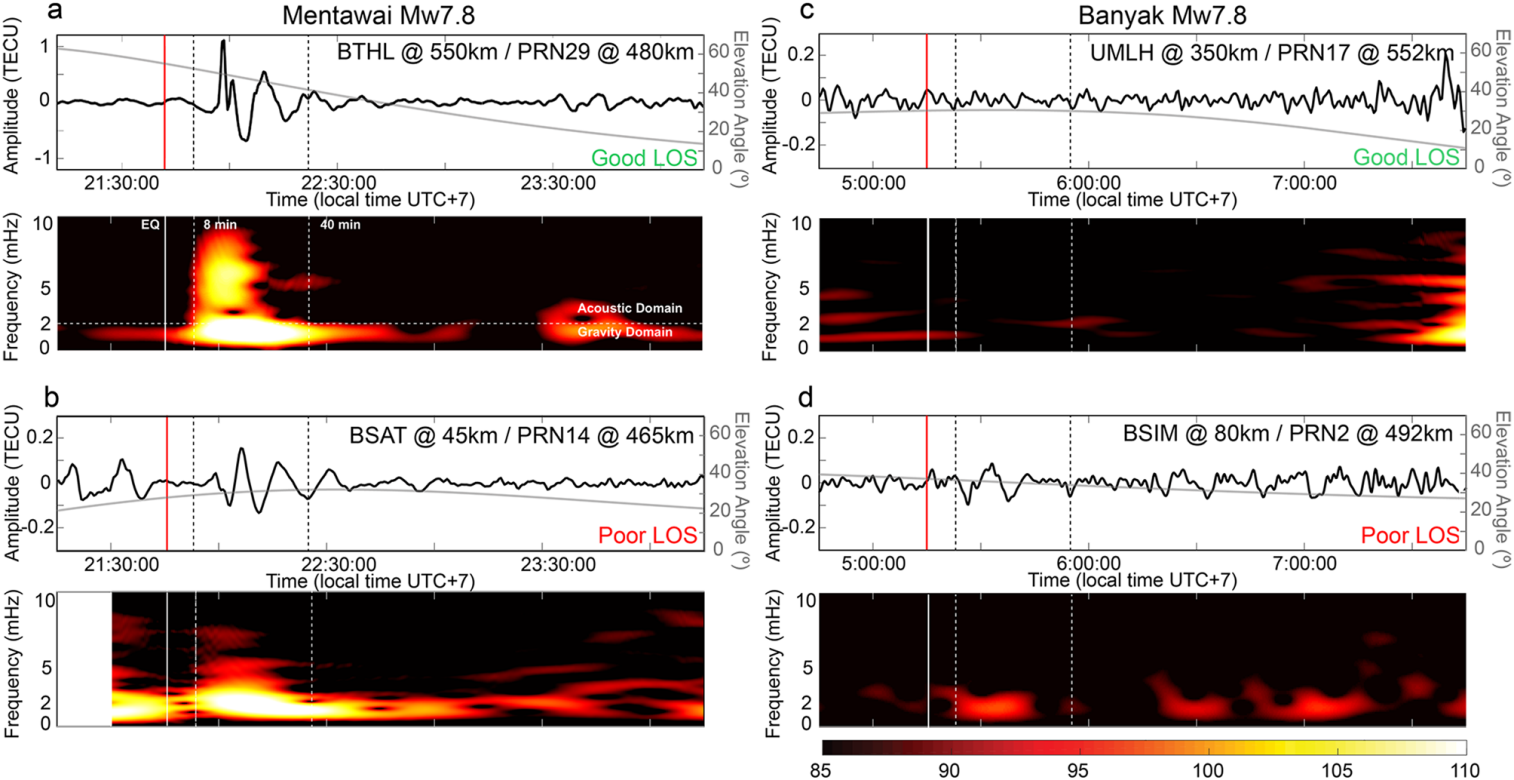
Filtered ionospheric TEC time series and related spectrograms extracted by (**a**) observations of satellite PRN29 with respect to station BTHL (**b**) observations of satellite PRN14 with respect to station BSAT at the time of the 2010 *M*_*w*_ 7.8 Mentawai earthquake (**c**) observations of satellite PRN17 with respect to station UMLH and (**d**) observation of satellite PRN2 with respect to station BSIM at the time of the 2010 *M*_*w*_ 7.8 Banyaks earthquake. Grey curves represent the elevation angle of the satellite-receiver ray path. Solid red and white vertical lines indicate the time of the events. Dashed vertical lines indicate the time of the first potential arrival of the AGW_epi_ (8 min) and the minimum time of IGW_tsuna_ observation (40 min) in the ionosphere. Horizontal dashed lines are the Brünt-Vaïsalla frequency that represents the limit between gravity and acoustic domains.

Accordingly, to be able to compare the TEC signatures of the two events analyzed in this work, we selected (from Fig. 2) four satellite-station pairs (Fig. 3) with similar angles and distances from the epicenter area that better represent the different LOS scenarios: Good LOS, with receiver-satellite pairs BTHL-PRN29 and UMLH-PRN17 for the Mentawai and Banyak Islands respectively; and Poor LOS, with BSAT-PRN14 and BSIM-PRN2 for the Mentawai and Banyak Islands respectively. During the Mentawai event the TEC shows a perturbation related to the AGW_epi_ (Fig. 2) with a frequency signature between 1 and 7 mHz (Fig. 3a,b) appearing 8 min after the main shock (21:42:23 local time, UTC + 7) and propagating away from the epicenter at a horizontal speed of 600–800 m/s (Fig. 2c). Later, a second weaker TEC perturbation at a lower frequency (~ 1.5 mHz), related to the IGW_tsuna_ appears to travel at a horizontal speed of ~ 250 m/s (Fig. 2d), consistent with the tsunami speed of ~ 220 m/s observed by DART buoy 56,001, located ~ 1,600 km from the epicenter^6^. The arrival time of IGW_tsuna_ observed at the distance of 480 km from the epicenter (Fig. 3a) is coherent with the tsunami propagation. A much smaller TEC perturbation followed the Banyaks earthquake (Figs. 2e, 3c,d), which generated a much weaker tsunami that did not cause any damage (see “Material and methods" section).

Figure 3 shows that regardless the LOS scenario, the Mentawai earthquake induces a greater signature in the GNSS-TEC highlighting the important amount of energy transferred in the ionosphere by this event. On the contrary for the Banyaks earthquake the TEC signature remains very weak also with a Good LOS geometry. Indeed, the GNSS-TEC traces in the hodochrones in Fig. 2c,f show that the satellite coverage is dense enough to assure the favorable observation of the AGW_epi_ and avoid attenuation effects related to the GNSS LOS geometry^22^.

**Figure 4:**
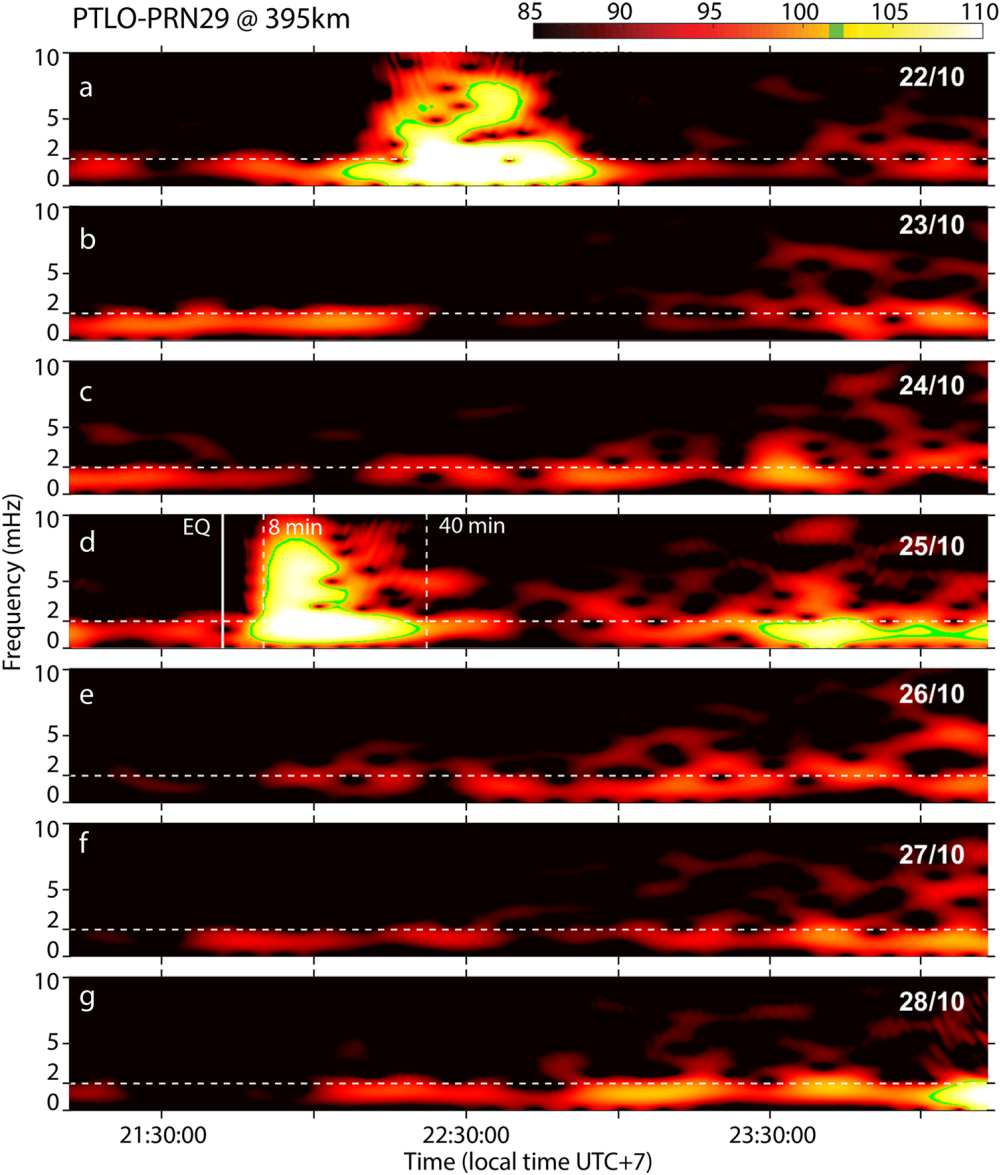
Spectrograms of the filtered TEC signal recorded by the pair of station PTLO and satellite PRN29 at the epicenter of the 2010 *M*_*w*_ 7.8 Mentawai earthquake over seven consecutive days. Horizontal dashed lines are the Brünt-Vaïsalla frequency that represents the limit between the gravity and the acoustic domain. Vertical lines in the central panel (**d**) indicate, respectively from left to right: the time of the event (solid line); the first potential arrival of the AGW_epi_ (8 min, dashed line); the minimum time of IGW_tsuna_ observation (40 min, dashed line) in the ionosphere. Green contour lines mark where the intensity of the signal is above a threshold value that represents the mean background level (MBL) calculated on quiet days.

**Figure 5:**
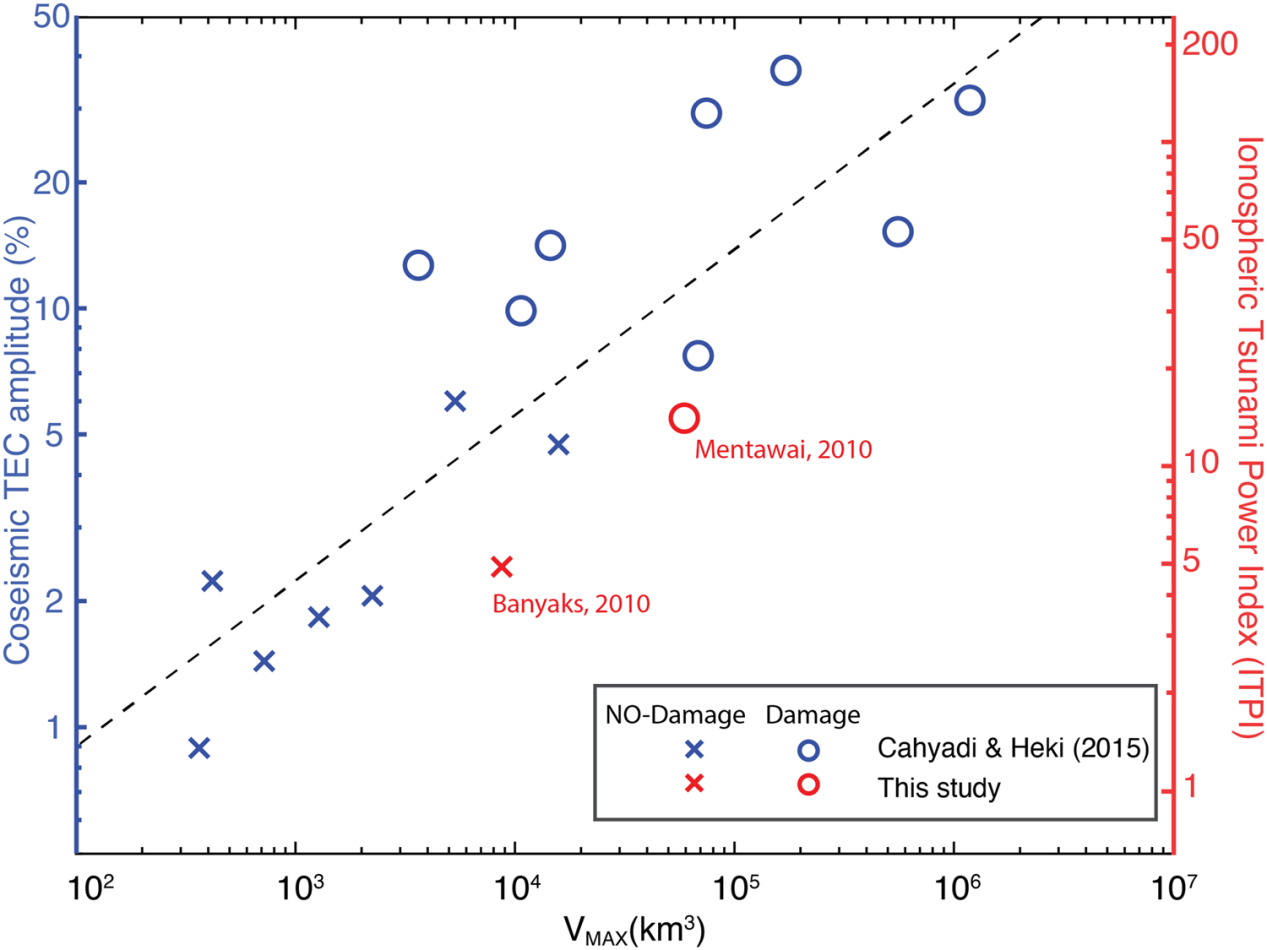
Relation between seafloor maximum volume displaced (V_max_), coseismic TEC amplitude and ITPI for 17 events. Blue symbols represent earthquakes from the literature^22,24,25^. Red symbols represent the Mentawai and Banyak earthquakes discussed in this paper for which both the coseismic TEC amplitude and the ITPI have been computed. The left axis represents the TEC coseismic amplitudes normalized by background vertical TEC estimated from Global Ionospheric Maps (GIM) as defined by Cahyadi and Heki^22^. The right axis represents the ITPI for the Mentawai and Banyak events scaled proportionally to the computed coseismic TEC amplitude. X symbols represent events that generated a very small tsunami that did not cause damage. O symbols represent events that did generate a tsunami and damage.

Spectral analysis (Fig. 4) of observed TEC during the days before and after the Mentawai event reveals the unique characteristics of the AGW_epi_ compared to the mean background level (MBL). The Ionospheric Tsunami Power Index (ITPI) is here introduced to define the ratio between TEC perturbation and MBL in order to remove the ionospheric dynamics, and consequently be able to compare the ITPI computed in different ionospheric conditions (see “Material and methods" section). We highlight that the ITPI is related to the entire spectrum of energy of the AGW_epi_ and consequently provides an estimate of the strength of a perturbation detected in the ionosphere. Compared to the dTEC amplitude estimation used by previous authors, our approach is not affected by frequency pass-band filtering or polynomial fit to obtain the perturbation of TEC. The ITPI for the Mentawai event was 14, as TEC observations revealed a perturbation 14% larger than the MBL (Fig. 4). A comparable energetic signature related to external localized phenomena (e.g., plasma bubbles, traveling ionospheric disturbances) appeared on 22 October 2010 but this was not related to a seismic event and consequent tsunami early warning, showing the importance to couple ionospheric observations with conventional techniques. Weaker TEC perturbations followed the Banyaks earthquake, at approximately 5% of the MBL (Supplemental Fig. 3), confirming the presence of a weaker tsunami (ITPI = 5). The ITPI measured for the two events shows a correlation with the values of seafloor uplift reported by Cahyadi and Heki^22^, by the United States Geological Survey (USGS, https://earthquake.usgs.gov/earthquakes/), and by Hill et al.^20^. We complete our observations for the Mentawai and Banyak Islands with TEC observations for additional 17 tsunamigenic events extracted by Cahyadi and Heki^22^ and listed in Table 1. We correlated the TEC amplitude (blue symbols in Fig. 5) with the seafloor uplift and, for comparison, with our new ITPI. To better quantify the contribution of the seafloor uplift, we introduce a weighting factor here referred as the seafloor maximum volume displaced (V_max_). This parameter is obtained from the product of the maximum seafloor uplift and the rupture area (A) (see “Material and methods" section). Seafloor uplift is in reality quite spatially variable, often poorly constrained by slip inversions, and likely to be supplemented by inelastic uplift that is not accounted for in most models. However, this parameter can provide a first-order proxy for comparison with the ITPI. To emphasize the originality of our work we highlight that Cahyadi and Heki^22^ correlate the TEC (%) perturbations with the moment magnitude of the events and not with the consequent seafloor maximum volume displaced (V_max_), which takes into account also the extent of the seafloor uplift directly related to the tsunami genesis. Our empirical model, showed in Fig. 5, supports the possibility of obtaining information about the tsunami genesis from ionospheric perturbations.

**Table 1:** List of the events discussed in Fig. 5.

Date	Location	Mw	Max uplift (m)	A (km^2^)	V_max_ (km^3^)	Max water height (m)	Damage	Loss
04/10/94	Hokkaido-toho-oki	8.3	2^b^	5,400	1.08E+4	10.40	Yes ~ $1M	–
25/09/03	Tokachi-oki	8	0.88^a^	78,720	6.93E+4	4. 40	Yes ~ $1M	–
05/09/04	Kii-hanto-oki(fore)	7.1	0.3^b^	2,400	7.20E+2	0.63	No	–
05/09/04	Kii-hanto-oki	7.4	0.7^b^	3,200	2.24E+3	0.93	No	–
26/12/04	Banda Aceh	9.2	3.4^a^	240,000	1.2E+6	50.90	Yes ~ $10B	227,899
03/05/06	Tonga	7.9	0.53^a^	10,075	2.41E+3	0.27	No	–
15/11/06	Central Kuril	8.2	0.46^a^	32,000	7.38E+3	21.90	Yes ~ 1-$5 M	–
16/07/07	Chuetsu-oki	6.6	0.3^b^	1,213	3.64E+2	Few cm	No	–
12/09/07	Sumatra Bengkulu	8.5	1.08^a^	70,000	7.56E+4	5	Yes ~ $1M	–
12/09/07	Sumatra Benglulu(after)	7.9	0.75^a^	21,120	1.58E+4	–	No	–
15/07/09	New Zeland	7.8	1.15^a^	3,200	3.68E+3	0.47	Yes	–
27/02/10	Maule	8.8	2.9^a^	60,000	1.74 E+5	29	Yes ~ $30B	153
06/04/10	Banyaks	7.8	0.42^a^	20,736	8.71E+3	0.44	No	–
25/10/10	Mentawai	7.8	1.9^c^	31,500	5.39E+4	16.9	Yes ~ $39M	431
09/03/11	Tohoku-oki(fore)	7.3	0.17^a^	2,457	4.18+2	0.60	No	
11/03/11	Tohoku-oki	9	5.27^a^	107,100	5.64E+5	38.90	Yes ~ $220B	18,434
28/10/12	Queen Charlotte Islands	7.8	0.32^a^	4,200	1.3E+3	12.98	No	–

Values of Max water height, Damage, and Loss are from NOAA (https://ntwc.ncep.noaa.gov/).

*Mw* moment magnitude, *A* rupture area, *V*_*max*_ seafloor maximum volume displaced.

^a^Obtained from (USGS).

^b^Values from (Cahyadi and Heki^22^).

^c^Value from (Hill et al.^20^).

Coupled with early seismic observations of an earthquake (estimating magnitude and area of slip), the detection of the AGW_epi_ by GNSS-TEC could provide valuable additional information—at the optimistic scenario of 8 min following the initiation of earthquake rupture—on the level of seafloor uplift and thus the potential of a significant tsunami. We highlight that the amplitude of the ionospheric TEC perturbation induced by the AGW_epi_ is directly related to the seafloor maximum volume displaced (V_max_) (Fig. 5) and we do not need to intermediately evaluate the magnitude of the event or the source parameters to estimate the tsunami risk. Additionally, estimation of GNSS high-rate position has been demonstrated as a valid technique for source estimation and tsunami risk evaluation to complement seismic networks (e.g.,^5^). Thus, we can use the same GNSS receivers for the source estimation and the consequent early tsunami potential evaluation using positioning techniques and validate it using the ITPI analysis. We can confirm the uplift and related tsunami risk by looking for the TEC perturbation induced by the AGW_epi_ within 8 min after the mainshock. After 40 min the TEC perturbation induced by the IGW_tsuna_ gives a direct estimate of the oceanic displacement induced by the tsunami^17^ propagating in the open ocean. The IGW_tsuna_ signature in the TEC has been already observed in real-time^23^, proving the potential role of ionospheric monitoring in real-time early warning.

With this work, we prove the maturity of ionospheric observations for evaluating tsunami potential, further showing the capability to discriminate the tsunamigenic nature of slow rupture events characterized by a low level of seismic energy radiation. In particular, estimations of ITPI could be fed into existing tsunami warning systems as an additional piece of information to alert for the presence of anomalous tsunami earthquakes and/or guard against false warnings. We hope to open a new paradigm in tsunami warning systems, based on the redundancy of information from the synergy of classic and new techniques to reduce damages and loss of life.

## Material and methods

To investigate the TEC variations caused by the 2010 Mw 7.8 Mentawai and Banyaks earthquakes, we analyzed 15-s data from 29 and 25, respectively, continuous GNSS stations of the SuGAr network that were in operation on the day of each earthquake (Fig. 2). We analyzed the GNSS data for the week centered on the day of each earthquake to obtain the ionospheric perturbations before, during, and after the events.

We calculated the TEC by applying a method similar to the one described by Calais and Minster^26^. To obtain a more accurate measure of the apparent distance between a satellite and receiver, we used the carrier phase (L1 & L2), neglecting the less precise pseudo-range measurements (P1 & P2):

1 $$TEC=\frac{(L1-L2)}{40.3} \frac{{f}_{1}^{2}{f}_{2}^{2}}{{f}_{1}^{2}-{f}_{2}^{2}}$$

where, *f*_*1*_ and *f*_*2*_ are, respectively, the corresponding high and low GPS frequency and TEC is the total electron content measured in TEC units (1 TECU = 10^16^ el/m^2^). We detected and corrected the cycle slips using the program *Ninja* within the GPS-Inferred Positioning System and Orbit Analysis Simulation Software (GIPSY-OASIS) version 6.2^27^.

As a consequence of the integrated nature of TEC, the observed ionospheric perturbations result from a large range of altitudes from the satellite to the receiver. However, the main contribution to the observed TEC variations is located around the height of the maximum of ionosphere ionization (the F2 layer). For this study, we fixed the altitude of the F2 layer at 300 km, which can be considered as the average altitude for day and night for the Equator. The intersection of the line of sight between each GNSS satellite-receiver pair at this altitude is termed the ionospheric pierce point (IPP), which we use to spatially visualize the observed TEC perturbations. In order to identify perturbations caused by the earthquake and the subsequent tsunami, we filtered all the initial TEC time series on a defined range of frequencies following the same methodology as Rolland et al.^28^. We applied a 1 to 10 mHz bandpass finite impulse response (FIR) Butterworth filter in order to remove the contributions from daily ionospheric variabilities, satellite motions, and instrumental biases.

We introduce for the first time the Ionospheric Tsunami Power Index (ITPI) to rapidly identify tsunamigenic earthquakes:

2 $$ITPI=\left(\frac{{PSD}_{MAX}}{MBL}-1\right) \cdot 100.$$

The ITPI corresponds to the ratio between the maximum level of power spectral density of the TEC (PSD_MAX_) recorded during the event, and the mean background level (MBL; defined as the maximum level of PSD recorded during the 6 days preceding the earthquake, averaged by the number of days). By averaging the background noise for several days prior to the event, we are able to remove outliers and smooth the noisy parts related to other perturbations traveling in the ionosphere. This new parameter could allow us to automatically identify the seafloor uplift and the consequent tsunamigenic potential of an event (Fig. 5). The relation for the Mentawai and Banyak events can be observed in Fig. 5, which shows, on the right side, the ITPI scale for the two events. The same relation with the seafloor maximum volume displaced V_max_ can be observed for the Coseismic Ionospheric Disturbance (CID), which is shown on the left side for both the Mentawai and Banyak events and 17 earthquakes described in the literature^22^ and listed in Table 1. To calculate the seafloor maximum volume displaced (V_max_), we multiply the maximum uplift value for the rupture area (A). Values of uplift are provided by the US Geological Survey (USGS, https://earthquake.usgs.gov/earthquakes/) resulting from finite fault, calculated using Okada-style deformation codes^29^. We highlight that the ITPI is a more accurate way to estimate the ionospheric perturbation as it is calculated in the spectral domain and it is consequently free of eventual pass-band filter applied to CID.

### Tsunami warning response to the two Mw 7.8 Mentawai and Banyaks earthquakes

We analyzed two *M*_*w*_ 7.8 earthquakes (Fig. 2) that occurred in 2010 along the Sunda megathrust (Indonesia): The 6 April Banyaks earthquake^18,19^ and the 25 October Mentawai earthquake^7,20,21^. Although they had the same magnitude, only the Mentawai earthquake produced an anomalously large tsunami.

The Banyaks earthquake ruptured a deeper portion (20–30 km) of the megathrust, producing relatively small uplift of the seafloor and thus only a small tsunami (max water height: 44 cm, max run up: 6 m, source NOAA database). On the other hand, the Mentawai earthquake was a shallow event with estimated slip concentrated at depths of < 6 km, no more than ~ 50 km away from the trench. Given the lower rigidity of the shallow sediments at this location, the Mentawai event generated considerably higher levels of slip^20,30^ than the Banyaks event^18^ while maintaining the same moment magnitude. The reported shake intensity at 150 km distance from the epicenter was MMI 5 and MMI 6 for the Mentawai, and for the Banyaks events, respectively. The high slip at shallow depths resulted in seafloor uplifts of up to several meters, producing the outsize tsunami with a reported maximum run up > 16 m^20^.

For the Mentawai event, the Badan Meteorologi Klimatologi Dan Geofisika (BMKG) of Indonesia initially estimated the magnitude as 7.2 and issued a tsunami warning within 5 min of the earthquake. The warning was afterward cleared without receiving information of tsunami damage^21^. The German Indonesian Tsunami Early Warning System (GITEWS) recorded the tsunami on a surface GPS buoy located in the Mentawai islands (GITEWS SUMATRA-03) within 10 min of the earthquake, with amplitudes of ~ 15 cm and a period of a few minutes^21,30^. Later the tsunami arrival was recorded by the Padang tide gauge about 1 h after the earthquake, and by the DART station 56,001 located around 1,600 km southeast of the epicenter about 2 h after the earthquake^21,30^. However, no tsunami warning was issued because some buoys had been vandalized^31^ and were not in operation. For the Banyaks event, BMKG also recognized a tsunami potential and immediately issued a tsunami warning that was canceled later, as only a minor tsunami was generated. For both events, the Pacific Tsunami Warning Center (PTWC) did not issue a warning, but a less severe tsunami watch, which was also canceled later (~ 2 h after). The GEOFON global seismological broadband network operated by the German GeoForschungsZentrum (GFZ) produced a real-time model for the two earthquakes, estimating a magnitude of *M*_*w*_ 7.6 for the Banyaks event and a magnitude of *M*_*w*_ 7.8 for the Mentawai event.

The Mentawai event did not provide a natural warning: witnesses reported feeling only weak, long-period shaking^20^. While many of the people on the islands knew to evacuate to higher ground on feeling strong earthquake shaking, they did not recognize this event was so dangerous. The characteristics of tsunami earthquakes make them particularly dangerous and exemplify the need for additional warning systems.

### The current state of the art for tsunami early warning

Due to difficulties in accuractely estimating the source extent, conventional approaches – such as seismological methods—encounter difficulties in estimating the tsunamigenic potential of an earthquake. This problem has been addressed by various studies. Convers and Newman^32^ developed a new method to rapidly discriminate between normal and slow ruptures events, such as those of tsunami earthquakes^3,7^. Kanamori and Rivera^2^ developed a method based on W-phases to determine seismic source parameters for tsunami warning purposes. This method can be used to reliably determine magnitude within ~ 20 min of the event^33^. Recent studies have shown that real-time GPS and seismic data can also be used in a combined approach^5^. This technique allows for rapid determination of the size of the source within few minutes from the rupture^34^, resulting in a more effective analysis compared to the seismic analysis alone, which suffers from saturation at large magnitudes. Melgar et al.^34^ tested their algorithm for rapid magnitude estimation with high-rate GPS data for a large number of events, with the Mentawai event being the least successful one. The uncertainties in the magnitude estimation for this particular event highlight again that additional techniques for rapid identification of tsunami earthquakes could improve current warning systems.

Several techniques that imply direct approaches, such as the use of offshore instruments, have been extensively applied for tsunami detection. NOAA developed and deployed the Deep-ocean Assessment and Reporting of Tsunami (DART). DART consists of a sea floor bottom pressure recorder and a moored surface buoy (https://www.ndbc.noaa.gov/dart/dart.shtml). Nevertheless, a DART buoy is primarily suitable to detect a far-field tsunami, as they are deployed and anchored at least 250 miles away from the shore^33^ thus being less useful for near-field events. GPS buoys and tide gauges, such as these deployed by the Nationwide Ocean Wave information network for Ports and HArbourS (NOWPHAS) near the Japanese coastline, and by the GITEWS, which was operating along the Sumatran coast between 2005 and 2011^35^, have the potential to provide short time tsunami warning within 10 min of the earthquake. However, in addition to damage caused by weather conditions, floating buoys may be also subject to vandalism, particularly in marine water that is favorable to fishing^36^. Servicing damaged buoys can be challenging and costly.

Other ocean-bottom seismographic and tsunami observation systems, based on fiber-optics submarine cables, have also been developed for tsunami detection^37^. These techniques are based on monitoring seafloor earthquakes to detect tsunami. However, these systems come with deployment costs that are usually prohibitively high. More recently, Xerandy et. al.^38^ proposed a new method based on an underwater communication system composed of fiber optic cables and an undersea network of sensors that could give a 20-min warning time.

It is clear that any additional information that gives rapid identification of the tsunami potential resulting from an earthquake would be useful for the success of tsunami early warning systems. In this study, therefore, we analyze the ionospheric response to two test events of the same seismic magnitude, which provide a unique natural experiment to test if the ionosphere is sensitive to the rupture properties and tsunami genesis, and whether GNSS-TEC observations are able to identify the events that have a high level of tsunami potential.

## Supplementary information

Supplementary file1

## Data Availability

The datasets generated during and/or analysed during the current study are available in the DR-NTU repository, https://doi.org/10.21979/N9/3KUEM5.
