# Supplementary material for: Rapid identification of tsunamigenic earthquakes using GNSS ionospheric sounding
Source: Sci Rep. 2020 Jul 6;10:11054. doi: 10.1038/s41598-020-68097-w (PMC7338347; doi:10.1038/s41598-020-68097-w)
Supplement: Supplementary file 1 — Supplementary file1 [file 41598_2020_68097_MOESM1_ESM.pdf]

# Supplemental Figures for:

## Rapid identification of tsunamigenic earthquakes using GNSS ionospheric sounding

**Authors:** Fabio Manta[1,2,*], Giovanni Occhipinti[3,4], Lujia Feng[1], Emma M. Hill[1,2]

**Affiliations:**

[1]Earth Observatory of Singapore, Nanyang Technological University, Singapore.

[2]Asian School of the Environment, Nanyang Technological University, Singapore

[3] Université de Paris, Institut de Physique du Globe de Paris, CNRS, France.

[4]Institut Universitaire de France, France.

*Corresponding author, now at the IPGP[3]: manta@ipgp.fr

**This PDF file includes:**

**Supplemental Figure 1-3**

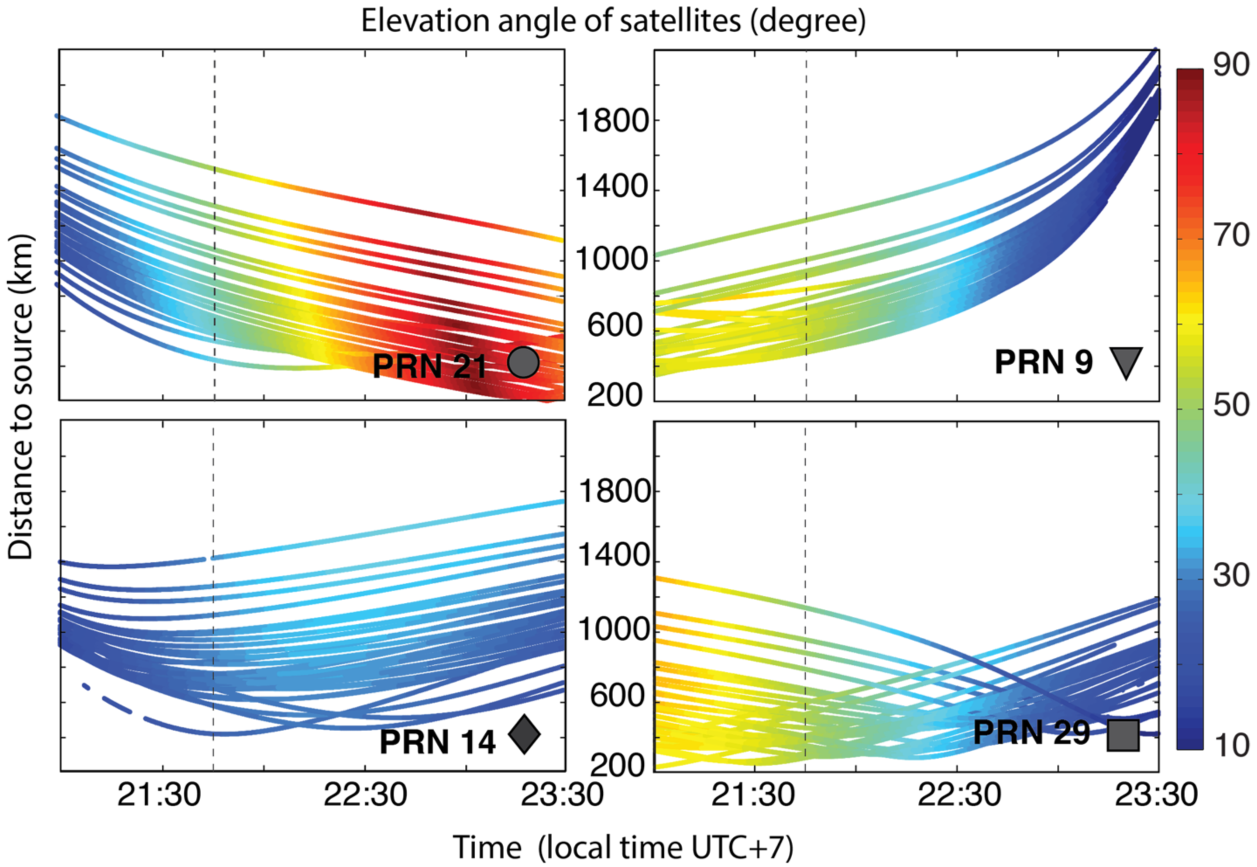


**Supplemental Figure 1.** Hodochrones showing the elevation angles related to the GPS satellites PRN 21, 9, 14 and 29, with respect to 29 stations available at the time of the Mentawai earthquake (local time: 21:42:23 UTC +7Vertical dashed lines represent the time of the event.

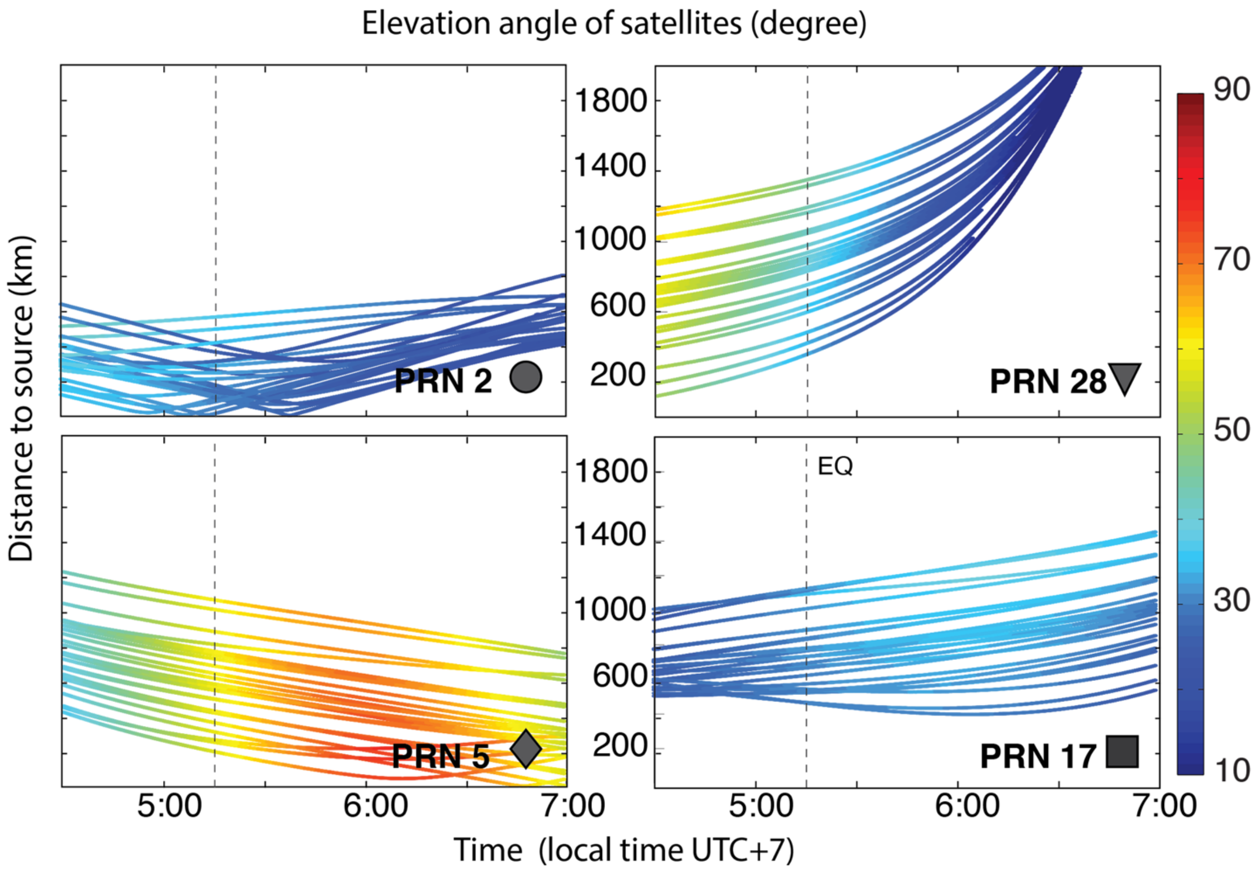


**Supplemental Figure 2.** Hodochrones showing the elevation angles related to the GPS satellites PRN 2, 28, 5 and 17, with respect to 25 stations available at the time of the Banyaks earthquake (local time: 5:15:02 UTC+7). Vertical dashed lines represent the time of the event.

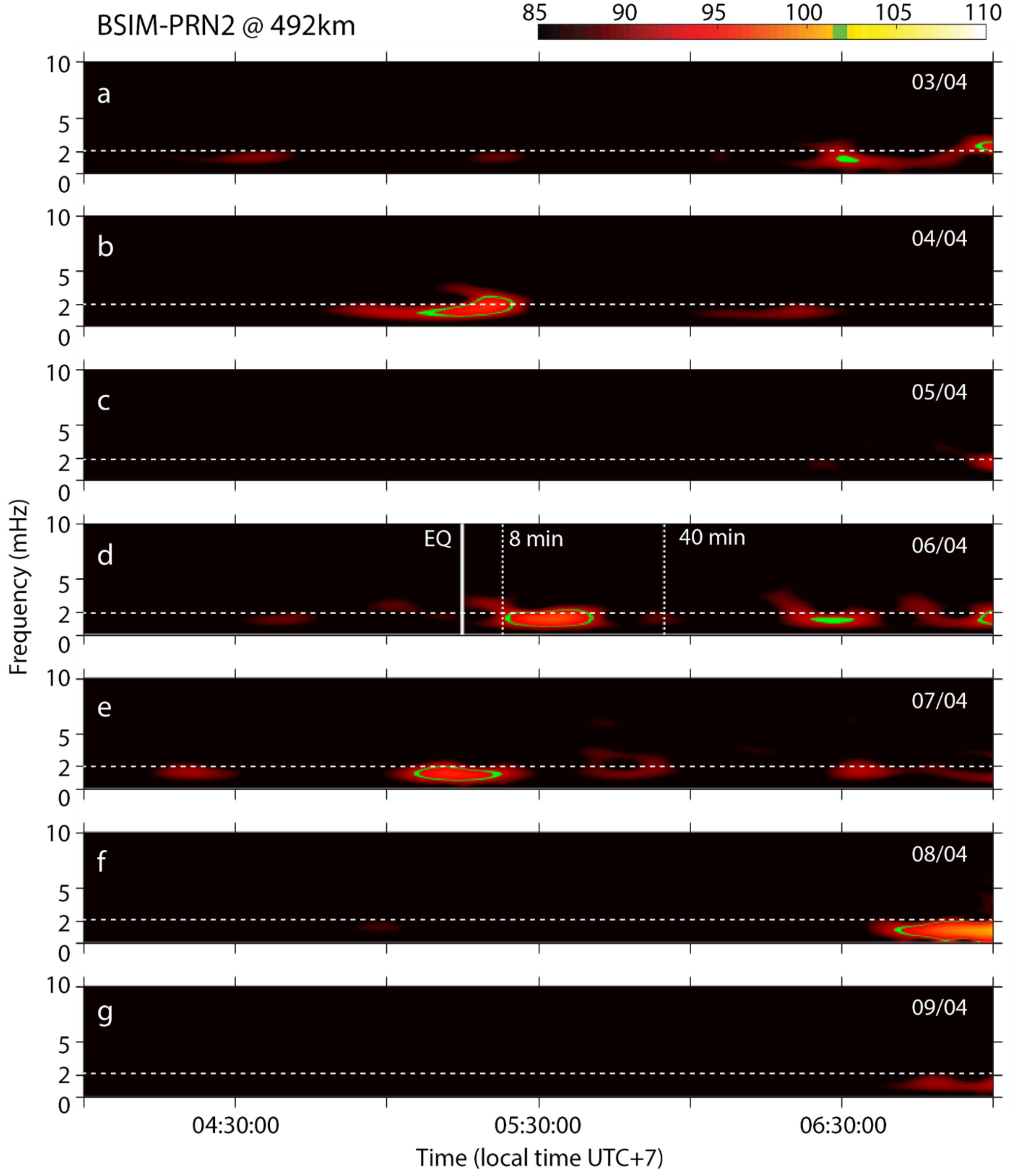


**Supplemental Figure 3.** Spectrograms of the TEC signal recorded by the same satellite-station pair at the epicenter of the 2010 Banyaks Mw 7.8 earthquake during 7 consecutive days. The horizontal dashed lines are the Brünt-Vaïsalla frequency that represents the limit between the gravity and the acoustic domain. Vertical lines in the central panel (d) indicate respectively from left to right: the time of the event (plain line); the first potential arrival of the AGWepi (8 min, dashed-line) and the earliest time of IGWtsuna observation (40 min, dashed-line). The green contour marks where the intensity of the signal is above a threshold value representing the mean background level (MBL).
